# Grants Recommended by the Distribution Committee

**Published:** 1914-12-26

**Authors:** 


### Grants Recommended by the Distribution Committee.

*The Distribution Committee desire to draw attention to the fact that it must not be assumed that the absence of a grant implies dissatisfaction.*

Alexandra Hospital for Children, £200.

Beckenham Cottage Hospital, £50. Belgrave Hospital, £850. Blackheath and Charlton Hospital, £100. Bolingbroke Hospital, £1,000. British Lying-in Hospital (hospital in Endell Street closed), a grant of £1,500 was set aside last year towards amalgamation with the Home for Mothers and Babies, Woolwich, in accordance with scheme to be approved.

Canning Town Women's Settlement Hospital (late Medical Mission), £450. Central London Ophthalmic Hospital, £250. Central London Throat and Ear Hospitals; capital grants amounting to £10,500 are set aside and promised for the purpose of an amalgamation of the Throat, Nose, and Ear Hospitals (see paragraph 6 of Report). Charing Cross Hospital, £4,000 of which £1,000 to new free wards in accordance with the scheme submitted to the Fund. Chelsea Hospital for Women, £500. Cheyne Hospital for Sick and Incurable Children, £200. City of London Hospital for Diseases of the Chest, Victoria Park, £3,500 of which £1,000 to improvements and £500 to erection of country sanatorium in accordance with the schemes submitted to the Fund. City of London Lying-in Hospital, £500. Clapham Maternity Hospital, £1,600 of which £1,500 to reconstruction in accordance with the scheme submitted to the Fund.

*Dreadnought* Hospital, £4,000 of which £500 to reduce debt.

East End Mothers' Lying-in Home, £200. East London Hospital for Children, £2,500 of which £500 to reduce debt. Eltham and Mottingham Cottage Hospital, £50.

Finchley Cottage Hospital, £25. Florence Nightingale Hospital for Gentlewomen, £350 of which £100 to reduce debt. French Hospital, £100. Friedenheim Hospital, £75.

General Lying-in Hospital, £200. German Hospital, £100. Gordon Hospital for Fistula, etc., £25. Great Northern Central Hospital, £4,500 of which £1,500 to reduce general fund debt. Grosvenor Hospital for Women, £350. Guy's Hospital, £7,500 of which £1,000 to building improvements in accordance with the scheme submitted to the Fund.

Hampstead General Hospital, £3,000 of which £1,100 to reduce general fund debt, and £150 as agreed upon amalgamation with North-West London Hospital. Home and Infirmary for Sick Children, £250. Home for Mothers and Babies, Woolwich, £150 to maintenance; a grant of £1,500 was set aside last year towards amalgamation with the British Lying-in Hospital in accordance with scheme to be approved. Hornsey Cottage Hospital, £25. Hospital and Home for Incurable Children, Hampstead, £50 in consideration of the fact that curable cases are admitted. Hospital for Consumption, Brompton (including sanatorium at Frimley), £3,000. Hospital for Diseases of the Throat, £900 maintenance grant, as agreed upon amalgamation with the London Throat Hospital (see paragraph 6 of Report). Hospital for Epilepsy and Paralysis, £1,000. Hospital for Sick Children, £3,500 of which £500 to reduce debt. Hospital for Women, Soho Square, £1,000. Hospital of St. John and St. Elizabeth, £350.

Infants' Hospital, £50. Italian Hospital, £200.

Kensington and Fulham General Hospital, £25. Kensington Dispensary and Children's Hospital, £25. King Edward Memorial Hospital, Ealing, £750 of which £350 to provision of children's ward in accordance with the scheme submitted to the Fund. King's College Hospital, £5,000 of which £3,000 to removal fund.

Leyton, Walthamstow, and Wanstead Children's and General Hospital, £400 of which £50 to provision of isolation ward in accordance with the scheme submitted to the Fund. London Fever Hospital, £550 of which £500 to rebuilding of isolation block in accordance with the scheme submitted to the Fund. London Homœopathic Hospital, £1,000 of which £250 to reduce general fund debt. London Hospital, £12,000. London Lock Hospital, £1,500 to the Harrow Road Hospital of which £500 to reduce general fund debt. London Temperance Hospital, £1,200. London Throat Hospital now amalgamated with the Hospital for Diseases of the Throat, Golden Square, which see; a maintenance grant of £900, as agreed, is made to the amalgamated hospital (see paragraph 6 of Report).

Maternity Charity and District Nurses' Home, Plaistow, £150. Memorial Hospital, Mildmay Park, £100. Metropolitan Hospital, £3,500 of which £500 to reduce general fund debt. Middlesex Hospital, £4,000. Mildmay Mission Hospital, £500. Miller General Hospital for South-East London, £2,250 of which £1,000 to reduce general fund debt.

National Dental Hospital now the Dental Department of University College Hospital, which see (see paragraph 7 of Report). National Hospital for Diseases of the Heart, £50. National Hospital for the Paralysed and Epileptic, £3,000 of which £500 to reduce debt. Nelson Hospital (late South Wimbledon, etc.), £250. New Hospital for Women, £500. Norwood Cottage Hospital, £50.

Paddington Green Children's Hospital, £1,000 of which £400 to reduce debt. Passmore Edwards Acton Cottage Hospital, £75. Passmore Edwards East Ham Hospital, £75. Passmore Edwards Hospital for Willesden, £25. Passmore Edwards Cottage Hospital, Wood Green, £50. Poplar Hospital, £250. Prince of Wales's General Hospital, £5,000 of which £2,000 to reduce debt.

Queen Charlotte's Lying-in Hospital, £750. Queen's Hospital for Children, £4,000 of which £2,000 is an exceptional grant to reduce debt.

Royal Dental Hospital of London, £400. Royal Eye Hospital, £350. Royal Free Hospital, £5,000 of which £2,500 to rebuilding of out-patient department in accordance with the scheme submitted to the Fund. Royal Hospital for Diseases of the Chest, City Road, £1,250. Royal Hospital, Richmond, £200. Royal London Ophthalmic Hospital, £2,250. Royal National Orthopædic Hospital, £2,500. Royal Waterloo Hospital for Children and Women, £1,750 of which £250 to reduce debt. Royal Westminster Ophthalmic Hospital, £50.

St. George's Hospital, £3,750. St. John's Hospital, Lewisham, £200. St. Luke's House, £25. St. Mark's Hospital, £200. St. Mary's Hospital, £3,500. St. Mary's Hospital for Women and Children, Plaistow, £750. St. Monica's Home Hospital, £50. St. Peter's Hospital for Stone, £300 of which £200 towards the erection of fire escape staircases. St. Saviour's Hospital, Osnaburgh Street, £150. Salvation Army Maternity Hospital, £50. Samaritan Free Hospital, £1,250 of which £250 to sanitary improvements. Santa Claus Home, £50. Stoke Newington Home Hospital for Women, £25.

University College Hospital, £3,800; the Fund is glad to note the successful issue of the negotiations for amalgamation with the National Dental Hospital (see paragraph 7 of Report).

Victoria Hospital for Children, £600.

West End Hospital for Diseases of the Nervous System, £250. Western Ophthalmic Hospital, £500 to reduction of debt on rebuilding of out-patient department. West Ham and Eastern General Hospital, £2,500. West London Hospital, £3,000. Westminster Hospital, £4,000 of which £1,000 to reduce debt. Wimbledon Hospital, £100. Woolwich and Plumstead Cottage Hospital, £25.

**Total Grants Recommended to Hospitals for the Year 1914 ... ... ... £133,500**

